# Quantitative analysis of axonal fiber activation evoked by deep brain stimulation via activation density heat maps

**DOI:** 10.3389/fnins.2015.00028

**Published:** 2015-02-10

**Authors:** Christian J. Hartmann, Ashutosh Chaturvedi, J. Luis Lujan

**Affiliations:** ^1^Department of Biomedical Engineering, Cleveland Clinic FoundationCleveland, OH, USA; ^2^Department of Neurology, Medical Faculty, Heinrich-Heine University DüsseldorfDüsseldorf, Germany; ^3^Medical Faculty, Institute of Clinical Neuroscience and Medical Psychology, Heinrich-Heine-University DüsseldorfDüsseldorf, Germany; ^4^Department of Biomedical Engineering, Case Western Reserve UniversityCleveland, OH, USA; ^5^Department of Neurologic Surgery, Mayo ClinicRochester, MN, USA; ^6^Department of Physiology and Biomedical Engineering, Mayo ClinicRochester, MN, USA

**Keywords:** deep brain stimulation, cortical excitability, axonal activation, computational model, individualized medicine

## Abstract

**Background:** Cortical modulation is likely to be involved in the various therapeutic effects of deep brain stimulation (DBS). However, it is currently difficult to predict the changes of cortical modulation during clinical adjustment of DBS. Therefore, we present a novel quantitative approach to estimate anatomical regions of DBS-evoked cortical modulation.

**Methods**: Four different models of the subthalamic nucleus (STN) DBS were created to represent variable electrode placements (model I: dorsal border of the posterolateral STN; model II: central posterolateral STN; model III: central anteromedial STN; model IV: dorsal border of the anteromedial STN). Axonal fibers of passage near each electrode location were reconstructed using probabilistic tractography and modeled using multi-compartment cable models. Stimulation-evoked activation of local axon fibers and corresponding cortical projections were modeled and quantified.

**Results**: Stimulation at the border of the STN (models I and IV) led to a higher degree of fiber activation and associated cortical modulation than stimulation deeply inside the STN (models II and III). A posterolateral target (models I and II) was highly connected to cortical areas representing motor function. Additionally, model I was also associated with strong activation of fibers projecting to the cerebellum. Finally, models III and IV showed a dorsoventral difference of preferentially targeted prefrontal areas (models III: middle frontal gyrus; model IV: inferior frontal gyrus).

**Discussion**: The method described herein allows characterization of cortical modulation across different electrode placements and stimulation parameters. Furthermore, knowledge of anatomical distribution of stimulation-evoked activation targeting cortical regions may help predict efficacy and potential side effects, and therefore can be used to improve the therapeutic effectiveness of individual adjustments in DBS patients.

## Introduction

It is hypothesized that the essential mechanisms underlying deep brain stimulation (DBS) of the subthalamic nucleus (STN) greatly rely on retrograde activation of corticofugal fiber pathways and subsequent modulation of cortical activity (Li et al., 2007, 2012; Dejean et al., 2009; Gradinaru et al., 2009).

However, the intensity and distribution of the downstream effects from stimulation of these fibers are currently not evaluated in clinical practice. Instead, a limited set of established stimulation settings is commonly tested for efficacy and side effects, since an extensive comparison of all potential combinations would go beyond the scope of clinical routine. But even with that restriction, current strategies to identify optimal stimulation effects rely on iterative, time-consuming adjustment of stimulation parameters (Volkmann et al., 2002). While such standard settings often lead to satisfactory effects, knowledge about cortical effects of STN DBS and their dependency on selected stimulation settings may help to find better, but normally untested stimulation adjustments. In addition, an advanced pre-selection of potentially advantageous stimulation settings would also expedite the process of identifying the best DBS set-up. Therefore, characterization of the interplay between DBS parameters, evoked cortical and subcortical activation patterns, and ensuing clinical effects is paramount to identifying DBS paradigms which provide optimal safety and efficacy.

In that regard, studies have shown that computational modeling of DBS can help identify axonal activation patterns associated with different targets and stimulation parameters using tractography activation models (TAM, Lujan et al., 2012, 2013). However, both the geometric complexity and broad distribution of modulated fiber pathways prevent the clinical use of this technique for quantitative evaluation of the network effects of stimulation. The present study introduces a quantitative method for TAM, which facilitates comparative analysis of axonal activation evoked by DBS across various cortical and subcortical areas. Consequently, it facilitates comparison of activation patterns across multiple stimulation settings and target locations, as shown in four models of STN DBS described below.

## Methods

### DBS models

**Figure 1:**
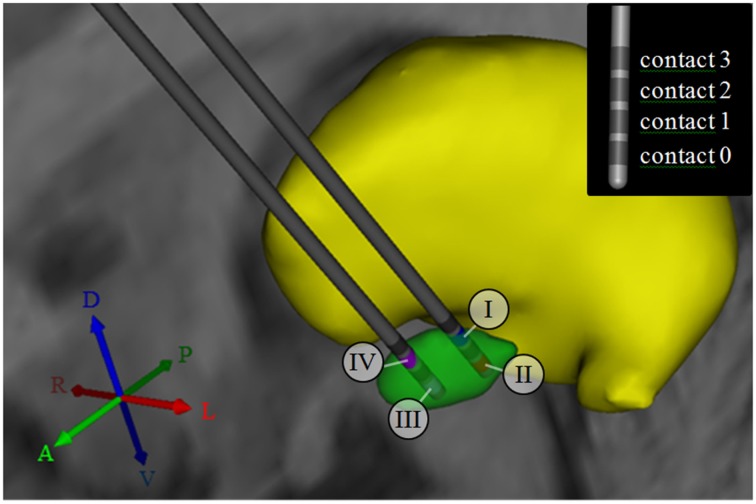
**3D anatomical relationship between subcortical nuclei, and active contacts for models I–IV**. The subthalamic nucleus (STN) is shown in green, while the thalamus is shown in yellow. For models I and II, the DBS electrode was placed in the posterolateral STN. For models III and IV, the electrode was shifted by 4 mm to a more anteromedial portion of the STN. The numbers I-IV indicate the selection of the active contact (cathode) for each of the four models. The inset in the upper right corner illustrates the consecutive numbering of the contact labels. Contact 2 was selected for monopolar stimulation in models I and IV, while contact 0 was selected for monopolar stimulation in models II and III. A, anterior; P, posterior; R, right; L, Left; D, dorsal; V, ventral.

**Figure 2:**
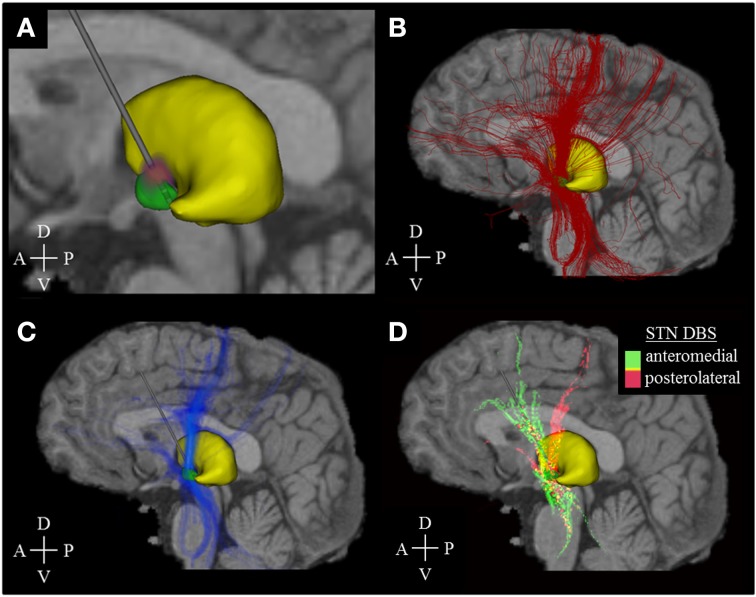
**Activation density heat maps**. All images are sagittal views from the left. The image sections **(A–C)** represent the activation results of model I only, while section D comprises the data of all models (I–IV). **(A)** Voltage distribution (red-purple) generated by DBS simulation (stimulation amplitude: −1.5 V, pulse width: 60 μ s, stimulation frequency: 130 Hz). Brighter colors correspond to higher voltage amplitudes. **(B)** Fiber pathways (red) activated by the electric field that was generated by the voltage distribution in **(A)**. **(C)** Activation density heat map, allowing for better identification of the main active fiber pathways. **(D)** Identification of active fibers pathways which are characteristic of a posterolateral (models I, II, pink color) or anteromedial (models III, IV, green color) DBS electrode placement. Yellow areas indicate an almost equal distribution between anteromedial and posterolateral stimulation. The associated mathematical operation is described in the running text. A, Anterior; P, Posterior; D, Dorsal; V, Ventral.

To characterize activation patterns across multiple stimulation settings and target locations, we used four stimulation models of left-hemisphere STN DBS based on a single brain template dataset (FMRIB Analysis Group, Oxford, UK) that included structural imaging (MP-Rage, 1 × 1 × 1 mm resolution) and diffusion tensor imaging (DTI, 2 × 2 × 2 mm resolution, 60 gradient directions, *b* = 1000 s/mm^2^). To create these four models, we first identified the location of the STN by co-registering the structural brain dataset with a 3D brain atlas using Cicerone (Miocinovic et al., 2007). Second, we placed a virtual stimulating electrode (Figure 1) in the posterolateral STN (models I and II) and within the anteromedial STN (models III and IV). The stimulating electrode was defined after a Medtronic 3389 DBS electrode (Medtronic, Minneapolis, MN) and consisted of four vertically aligned contacts with a length of 1.5 mm and an inter-contact distance of 0.5 mm (electrode diameter 1.27 mm, contact surface ~5.98 mm^2^). In models I and IV, stimulation was performed through contact 2 (Figure 1), which was placed at the dorsal border of the STN. In models II and III, stimulation was performed through contact 0, which was located toward the center of the STN, as opposed to its dorsal boundary. (Figure 1). Third, monopolar cathodic stimulation was applied to all models using −1.5 V, 60 μs pulses at 130 Hz. These stimulation settings were chosen to represent typical stimulation parameters to achieve a therapeutic effect in Parkinson's disease DBS. For each of the four models, a multi-resolution finite element method (FEM) model was constructed using COMSOL 3.1 (Comsol Inc., Burlington, MA) and SCIRun/Bio-PSE (Scientific Computing and Imaging Institute, University of Utah, Salt Lake City, UT) to describe the DBS electric field generated by monopolar cathodic stimulation (Butson et al., 2007). Accounting for the non-homogeneous anisotropic conduction characteristics of the brain, a conductivity tensor was calculated at each voxel of the DTI (Tuch et al., 2001). We solved for the voltage distribution in the tissue medium using the Poisson equation and included the FEM model and conductivity tensors (Figure 2A). These models included the set of stimulation settings, the capacitance of the electrode-tissue interface (3.3 μF), an encapsulation sheath (0.128 S/m), and a voltage drop of 42% (Miocinovic et al., 2006) in order to describe the tissue response to DBS (Chaturvedi et al., 2006, 2010) and to account for a representative impedance (1000 Ohm) measured from the DBS programming device (Butson et al., 2006).

### Probabilistic fiber model

A probabilistic model of axonal fibers was defined by following the four steps described below. We used FSL, a library of analysis tools for brain imaging data (Jenkinson et al., 2012), to identify 133,100 axonal trajectories. First, a Bayesian algorithm (bedpostX) was employed to estimate the diffusion parameters at each voxel of the DTI. Up to 2 fiber directions were calculated per voxel. Second, a cubic seed region within the DTI dataset was comprised by an 11 × 11 × 11 voxel cube (22 × 22 × 22 mm, 1331 voxels) centered on the midpoint of the active contacts for models I–IV. Third, each of the 1331 voxels within the seed region served as source to reconstruct 100 fibers with probabilistic tractography, respectively. For this purpose, the tool probtrackX iteratively created the 100 most likely individual streamlines with Euler's method, based on the diffusion parameters calculated with bedpostX (step length 0.5 mm, curvature threshold ±80°, maximum number of steps: 2000, termination of pathways that loop on themselves, and cerebrospinal fluid space as termination mask). Fourth, multi-compartment cable models of axons [5.7 μm diameter, 0.5 mm internodal distance (McIntyre et al., 2002)] were created using NEURON 7.0 (Hines and Carnevale, 1997) for each fiber pathway. The response of the 133,100 axon reconstructions to the electric field generated by DBS was simulated for each of the four models (McIntyre et al., 2004). An axon was determined to be active when a propagating action potential was induced (Figure 2B).

### Quantitative analysis of cortical activation

Novel activation density maps were developed to describe and quantify the distribution of DBS activated fibers pathways within a certain brain volume. These activation density maps were created by first overlaying a Cartesian grid (cell size of 1 × 1 × 1 mm) on the dataset encompassing all fiber geometries. Second, the number of active fibers passing through each grid cell, determined by the number of active fibers with at least one node of Ranvier within the cell boundaries, was used to describe the activation density within that cell. Third, activation density heat maps were created by converting each grid cell into a voxel within a 3D image. The intensity of each voxel was determined by the cell activation density. Fourth, primary active fiber pathways were identified by using intensity thresholding, which allowed for visual analysis of activation results (Figure 2C). The process itself was used for visualization only and was not applied for quantitative analyses as described below. It did not follow a strict mathematical regimen but was adjusted in a non-linear manual way to provide optimal visibility of the main pathways and was kept constant in the four models. Fifth, active pathways characteristic of posterolateral (models I and II) and anteromedial (models III and IV) DBS were calculated using the Hadamard product (element-wise multiplication of voxels): The activation heat map for model I was multiplied with the heat map of model II to explicitly highlight voxels with high active fibers densities in both models. Next, the activation heat map product of models III and IV was subtracted from the result of models I and II. Hence, positive voxel values indicated predominance of axonal pathways activated by posterolateral simulation, while negative values represented pathways characteristic of anteromedial stimulation. Sixth, color-coding was used to identify characteristic fiber pathways associated with each electrode placement (Figure 2D).

Seventh, multiple cortical regions (Table 1) were segmented from the structural imaging, using Freesurfer and based on the Desikan atlas, to quantify the active connections associated with each of the four models (Fischl et al., 2002; Desikan et al., 2006). The segmentation data were saved as three-dimensional label map with a voxel size that matched the cell dimensions of the grid (1 × 1 × 1 mm).

**Table 1:** **List of cortical regions of interest projected onto by fibers activated by the four models**.

**Frontal pole**	**Postcentral gyrus**	**Cingulate cortex**	**Temporal pole**
Orbitofrontal cortex	Supramarginal gyrus	Parahippocampal gyrus	Superior temporal gyrus
Superior frontal gyrus	Superior parietal cortex	Hippocampus	Middle temporal gyrus
Middle frontal gyrus	Inferior parietal cortex	Entorhinal cortex	Inferior temporal gyrus
Inferior frontal gyrus	Precuneus	Amygdala	Insula
Precentral gyrus	Occipital lobe	Fusiform gyrus	Cerebellum

*These regions were obtained from bilateral cortical segmentation*.

Eighth, the 3D cortical segmentations were interpolated onto the cell grid to allow definition of cortical regions with respect to the activation density heat maps. For this purpose, the label map was referenced to the structural imaging, the activation density heat maps, and the cell grid. Subsequently, each cell of the grid could be assigned to a distinct value of the label map, which in turn represented a cortical area of interest. To obtain the number connecting active fibers, all active fibers were counted, which intersected at least one of the associated cells of the grid. This number was calculated instead of summing up the activation densities of each associated cell to avoid over-representation of fibers coursing through multiple voxels within a target anatomical region. Fibers, which did not reach the cortical regions, were excluded from this quantitative analysis.

## Results

**Figure 3:**
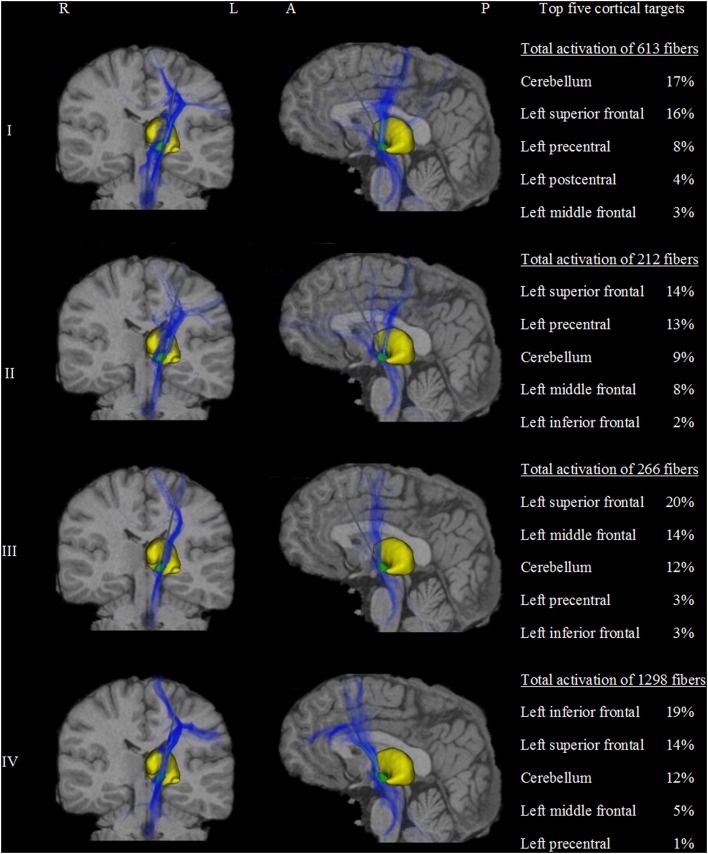
**Activation density heat maps showing axonal activation for the four models (I–IV)**. Left and middle columns show coronal and sagittal views, respectively. The subthalamic nucleus is shown in green and the thalamus in yellow. High densities of active fibers are associated with brighter color and less transparency. The major differences of activation results between the four models are clearly observed. Models I and II were highly connected to the precentral gyrus and the dorsal segment of the superior frontal gyrus. Additionally, model I showed strong cerebellar projections. In contrast, models III and IV showed stronger connections to prefrontal areas such as the anterior part of the superior frontal gyrus and the middle and inferior frontal gyri. Model III was associated with the middle frontal gyrus. Similarly, model IV presents a predominant connection to the inferior frontal gyrus. The right column shows a quantitative comparison of the anatomical distribution of active fibers between the four STN DBS models. The top five cortical targets including the percentage of active fibers targeting those regions are illustrated. R, right; L, left; A, anterior; P, posterior.

A total of 133,100 fibers were analyzed for each model. Fiber activation analysis suggested that a greater number of fibers were activated with DBS of the dorsal border of the STN, with 613 and 1298 active fibers for models I and IV, respectively. In contrast, DBS of the STN center resulted in lower fiber activation density, with 212 and 266 active fibers for models II and III. The frontal lobe and cerebellum were the main cortical targets in all models, with 36% (± 5%) and 13% (± 3%) of active fibers, respectively. Patterns of axonal activation and corresponding cortical projections for each of the four models are illustrated in Figure 3.

**Figure 4:**
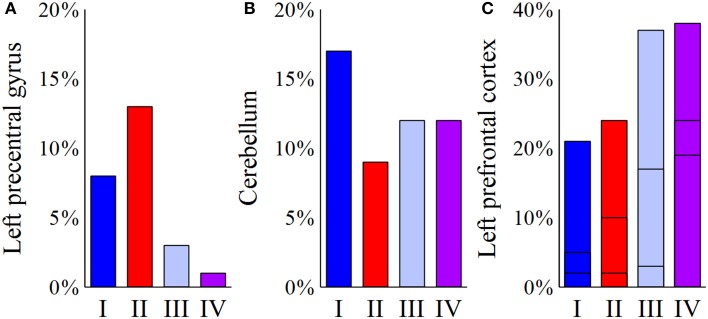
**Comparison of the four models (I–IV) with respect to the predominantly targeted cortical areas**. For each model, the bar plots represent the percentage of active fibers targeting a distinct region of interest. **(A)** Posterolateral stimulation (models I and II) present a considerable connection of active fibers to the left precentral gyrus, as opposed to anteromedial stimulation (models III and IV). **(B)** For the cerebellum, the highest percentage of connected active fibers can be found in model I. **(C)** For the left prefrontal cortex, the plotted bars are divided into three sections. The bottommost part reflects the inferior frontal gyrus, the middle part illustrates connections to the middle frontal gyrus, and the topmost part represents fibers targeting the superior frontal gyrus. While model III present relatively high percentages of fibers projecting to the left middle frontal gyrus, activation results for the left inferior frontal cortex show a clear predominance of model IV.

Despite multiple regions of common activation, marked differences between the models could also be observed. In our example data, posterolateral DBS (models I and II) was characterized by strong connections between the precentral cortex and the posterior part of the superior frontal gyrus, which can be functionally assigned to the primary motor cortex and the supplementary motor area, respectively. A characteristic feature of model I was the strong distribution of active connections to the cerebellum. Additionally, anteromedial STN DBS (models III and IV) was characterized by active fibers predominantly targeting the prefrontal cortex (anterior part of the superior frontal gyrus as well as middle and inferior frontal gyrus). While the superior frontal gyrus was a main target in both models, model III showed strong connections to the medial frontal gyrus, whereas model IV preferentially targeted the inferior frontal gyrus. A direct comparison of the four models with respect to the predominantly targeted cortical regions is provided in Figure 4.

## Discussion

In this study, we introduced a new method for quantitatively analyzing the effects of DBS therapy as an adjunct analysis technique for TAM. The method presented herein relies on tractography activation models (Lujan et al., 2013) and density activation heat maps to allow quantitative characterization and comparison of the effects of different stimulation parameters and targets on the pattern of cortical activation.

### Clinical implication

Our results showed that DBS-evoked activation depends on the electrode placement and choice of the active contact. However, activation density heat maps presented herein can also be applied to compare different stimulation settings like variations of the voltage or pulse width. Additionally, our heat map datasets facilitate mathematical and statistical analyses and therefore provide the option to compare activation patterns across a myriad of DBS targets and configurations. In models II and III, the active contact was located toward the center of the STN, which reduced activation density of white matter tracts surrounding it. In models I and IV, however, the active contacts were placed at the dorsal border of the STN in proximity to white matter tracts, resulting in a higher active fiber density. As a consequence, these modeling results suggest that stimulation at the dorsal border of the STN may require lower amplitudes, and hence lower battery consumption, to evoke a certain activation response.

Posterolateral placement of the DBS electrode within the STN (models I and II) is commonly selected for the treatment of advanced Parkinson's disease (Groiss et al., 2009). Our finding of high-density connections to cortical motor areas is supported by other studies providing evidence for cortical involvement in clinically effective DBS for treatment of Parkinson's disease (Hanajima et al., 2004; Karimi et al., 2008; Lai et al., 2014). Stimulation of the hyperdirect pathways is currently discussed to antidromically mediate these cortical effects (Li et al., 2014). Furthermore, DBS of the dorsal border of the posterolateral STN (model I)—potentially by modulation of adjacent structures like the zona incerta, the fields of Forel, and/or the pallidosubthalamic tract—has been suggested to provide superior symptom improvement. This especially applies for tremor reduction compared to stimulation deeper within the STN (Lanotte et al., 2002; Voges et al., 2002; Hamel et al., 2003; Herzog et al., 2004; Maks et al., 2009). In particular, stimulation of cerebellar connections of the zona incerta or parts of the cerebello-thalamic tracts has been associated with increased therapeutic efficacy (Herzog et al., 2007; Plaha et al., 2008). This finding was reproduced in model I, which predicted a high density of active fibers targeting the cerebellum. For future DBS programming, such predictions may help to additionally stimulate these areas in order to enhance clinical efficacy of the treatment.

In contrast, anteromedial placement of the DBS electrode within the STN (models III and IV) could be selected for the treatment of obsessive-compulsive disorder (Mallet et al., 2008; Chabardès et al., 2013). Clinically effective DBS was shown to alleviate functional abnormalities in the prefrontal cortex, which are associated with this disorder (Le Jeune et al., 2010). The distinct fiber pathways mediating therapeutic effects in anteromedial STN DBS for obsessive-compulsive disorder have yet to be determined. However, in analogy to STN DBS for Parkinson's disease, retrograde activation of so-called limbic hyperdirect pathways (Haynes and Haber, 2013) may be speculated to be important for a beneficial outcome (Nougaret et al., 2013). Complementary, our simulations demonstrated that prefrontal areas of the cortex are predominantly targeted by active fibers in models III and IV. In line with the dorsoventral organization of subcortical fibers targeting the prefrontal cortex (Lehman et al., 2011), stimulation via contact 2 mainly targeted basal parts of the prefrontal cortex, whereas stimulation using contact 0 focused on activating dorsolateral components.

### Current challenges, limitations, and opportunities

The activation density heat map approach presented herein exclusively focuses on cortical projections of fibers activated by DBS, and does not account for other attributes that might mediate its clinical efficacy, like restitution of exaggerated oscillatory synchronization as obtained from local field potential measurements (Kühn et al., 2008). However, this approach can substantially contribute to the identification of stimulation settings that provide an optimal combination of safety and therapeutic efficacy, particularly in cases where small changes in stimulation settings can result in significant differences in therapeutic improvement. Development of activation density heat maps is currently a time-consuming process, which prevents its current application in real-time. This limitation can be easily overcome by developing a database for a reference atlas brain that stores activation density heat maps for various stimulation settings and targets. Such an approach however would insert potential sources of error by not taking into account anatomical differences between individual patients. However, by combining this approach with artificial intelligence techniques, we could perform on-the-fly interpolation of different stimulation models; thereby increasing the usability of the technique in clinical practice. The creation of a reference atlas brain as a cross-sectional average of multiple subjects would also allow for statistical analyses like atlas-guided cluster analysis that would increase the integrity of reconstructed fiber pathways (Ros et al., 2013). Such an approach would reduce the risk of implementing fiber tracts that do not exist (false positives) and ensure that existing fiber tracts, which might not have been reconstructed in a single-subject analysis (false negatives), are incorporated. Additional information, like the average fiber diameter of distinct fiber tracts could be gathered from histological or imaging studies. Such data would help to account for structural heterogeneity of fiber pathways and therefore improve our simulation of the axonal response to DBS. In addition, colorization of fiber pathways that reach cortical areas of interest, in addition to quantitative comparison of cortical connections as introduced in this manuscript, would facilitate the visual comparison of activation results and the identification of crucial fiber pathways.

The method presented herein is a complementary tool to existing strategies for analyzing DBS-evoked changes of cortical activity. One of these strategies is functional imaging, which has been widely used to investigate the therapeutic and side effects of DBS (Albaugh and Shih, 2014). However, functional imaging techniques are not well suited to differentiate direct cortical activation resulting from polysynaptic network mechanisms via cortico-cortical or cortico-subcortical connections. In contrast, activation density heat maps are designed to explicitly identify axonal pathways and associated cortical projections directly affected by DBS (via antidromic or monosynaptic modulation). Electrophysiological studies have also been used to reveal the cortical effects of DBS (Devergnas and Wichmann, 2011; Walker et al., 2011, 2012; Whitmer et al., 2012). However, electrophysiological techniques are either invasive (electrocorticography) or limited by low spatial resolution (electroencephalography). Activation density heat maps are non-invasive and can provide good spatial resolution to allow identification of anatomical regions involved in the mechanisms of DBS. In combination with functional imaging and electrophysiological analysis, the activation density approach presented herein will improve differentiation between direct cortical effects and network mechanisms of DBS.

## Conclusion

Activation density heat maps are an adjunct analysis technique for TAM, which can help elucidate anatomical regions involved in the mechanisms of DBS. The predicted extent of fiber activation presented herein, which is supported by previous clinical findings, underlines the validity of our novel approach but also re-affirm that small differences in stimulation settings might result in clinically different outcomes. However, future studies should validate model predictions with additional clinical data. Prospective evaluation of this modeling approach in large patient populations will allow identification, analysis, and characterization of fiber tracts that mediate clinical efficacy. Furthermore, it will help predict and mitigate side effects associated with overstimulation and the use of sub-optimal stimulation parameter settings.

### Conflict of interest statement

Christian J. Hartmann received a research grant from the German Acadedemic Exchange Service (DAAD). Ashutosh Chaturvedi has intellectual property licensed to Boston Scientific. He also was a paid consultants for Boston Scientific. J. Luis Lujan has intellectual property licensed to Boston Scientific. The authors declare that the research was conducted in the absence of any commercial or financial relationships that could be construed as a potential conflict of interest.
